# Supplementary material for: Effectiveness of school-based interventions in delaying sexual debut among adolescents in sub-Saharan Africa: a protocol for a systematic review and meta-analysis
Source: BMJ Open. 2021 May 21;11(5):e044398. doi: 10.1136/bmjopen-2020-044398 (PMC8144038; doi:10.1136/bmjopen-2020-044398)
Supplement: Supplementary data [file bmjopen-2020-044398supp001.pdf]

Search strategy

Pubmed

1. (intervention[Title/Abstract]) OR (program*[Title/Abstract])
2. (school[Title/Abstract]) OR (institution[Title/Abstract]) OR (academic[Title/Abstract]) OR (education[Title/Abstract])
3. (sexual debut[Title/Abstract]) OR (sexual initiation[Title/Abstract]) OR (sexual delay[Title/Abstract]) OR (sexual activity [Title/Abstract])
4. (adolesce*[Title/Abstract]) OR ("young people"[Title/Abstract]) OR (youth[Title/Abstract]) OR (teenage*[Title/Abstract]) OR (learner[Title/Abstract]) OR (children[Title/Abstract])
5. (Africa south of the Sahara [MeSH Terms]) OR (Africa [MeSH Terms])
6. ("2009/01/01"[Date - Publication]: "2020/12/31"[Date - Publication])
7. #1 AND #2 AND #3 AND #4 AND #5 AND #6

   ("intervention"[Title/Abstract] OR "program*"[Title/Abstract]) AND ("school"[Title/Abstract] OR "institution"[Title/Abstract] OR "academic"[Title/Abstract] OR "education"[Title/Abstract]) AND ("sexual debut"[Title/Abstract] OR "sexual initiation"[Title/Abstract] OR "sexual delay"[Title/Abstract] OR "sexual activity"[Title/Abstract]) AND ("adolesce*"[Title/Abstract] OR "young people"[Title/Abstract] OR "youth"[Title/Abstract] OR "teenage*"[Title/Abstract] OR "learner"[Title/Abstract] OR "children"[Title/Abstract]) AND ("africa south of the sahara"[MeSH Terms] OR "africa"[MeSH Terms]) AND 2009/01/01:2020/12/31[Date - Publication]
