# Supplementary material for: Effectiveness of school-based interventions in delaying sexual debut among adolescents in sub-Saharan Africa: a protocol for a systematic review and meta-analysis
Source: BMJ Open. 2021 May 21;11(5):e044398. doi: 10.1136/bmjopen-2020-044398 (PMC8144038; doi:10.1136/bmjopen-2020-044398)
Supplement: Author's manuscript [file bmjopen-2020-044398.draft_revisions.pdf]

BMJ Open

BMJ Open is committed to open peer review. As part of this commitment we make the peer review history of every article we publish publicly available.

When an article is published we post the peer reviewers' comments and the authors' responses online. We also post the versions of the paper that were used during peer review. These are the versions that the peer review comments apply to.

The versions of the paper that follow are the versions that were submitted during the peer review process. They are not the versions of record or the final published versions. They should not be cited or distributed as the published version of this manuscript.

BMJ Open is an open access journal and the full, final, typeset and author-corrected version of record of the manuscript is available on our site with no access controls, subscription charges or pay-per-view fees (http://bmjopen.bmj.com).

If you have any questions on BMJ Open's open peer review process please email info.bmjopen@bmj.com

BMJ Open

Effectiveness of school-based interventions in delaying sexual debut among adolescents in sub-Saharan Africa: A protocol for a systematic review and meta-analysis

Journal:	*BMJ Open*
Manuscript ID	bmjopen-2020-044398
Article Type:	Protocol
Date Submitted by the Author:	07-Sep-2020

Complete List of Authors:	Maina, Beatrice; African Population and Health Research Center, Population Dynamics and Reproductive Health; University of the Witwatersrand Faculty of Health Sciences, School of Public Health<br>Juma, Kenneth ; African Population and Health Research Center, Population Dynamics and Sexual Reproductive Health<br>Igonya, Emmy; African Population and Health Research Center, PDRH<br>Osindo, Jane; African Population and Health Research Center<br>Wao, Hesborn; African Population and Health Research Center<br>Kabiru, Caroline; African Population and Health Research Center

Keywords:	PUBLIC HEALTH, Health policy < HEALTH SERVICES ADMINISTRATION & MANAGEMENT, Community child health < PAEDIATRICS

SCHOLARONE™
Manuscripts

I, the Submitting Author has the right to grant and does grant on behalf of all authors of the Work (as defined in the below author licence), an exclusive licence and/or a non-exclusive licence for contributions from authors who are: i) UK Crown employees; ii) where BMJ has agreed a CC-BY licence shall apply, and/or iii) in accordance with the terms applicable for US Federal Government officers or employees acting as part of their official duties; on a worldwide, perpetual, irrevocable, royalty-free basis to BMJ Publishing Group Ltd ("BMJ") its licensees and where the relevant Journal is co-owned by BMJ to the co-owners of the Journal, to publish the Work in this journal and any other BMJ products and to exploit all rights, as set out in our licence.

The Submitting Author accepts and understands that any supply made under these terms is made by BMJ to the Submitting Author unless you are acting as an employee on behalf of your employer or a postgraduate student of an affiliated institution which is paying any applicable article publishing charge ("APC") for Open Access articles. Where the Submitting Author wishes to make the Work available on an Open Access basis (and intends to pay the relevant APC), the terms of reuse of such Open Access shall be governed by a Creative Commons licence – details of these licences and which Creative Commons licence will apply to this Work are set out in our licence referred to above.

Other than as permitted in any relevant BMJ Author's Self Archiving Policies, I confirm this Work has not been accepted for publication elsewhere, is not being considered for publication elsewhere and does not duplicate material already published. I confirm all authors consent to publication of this Work and authorise the granting of this licence.

For peer review only

Target journal: BMJ open

Effectiveness of school-based interventions in delaying sexual debut among adolescents in sub-Saharan Africa: A protocol for a systematic review and meta-analysis

Short Title: School-based interventions and sexual debut

Authors: Beatrice W. Maina[1], Kenneth Juma[1], Emmy Kageha Igonya[1], Jane Osindo[1], Hesborn Wao[1], Caroline W. Kabiru[1]

Affiliation

[1]African Population Health and Research Center, Nairobi, Kenya

Corresponding Author

Beatrice W. Maina

Email: bmaina@aphrc.org

P.O. Box 10787, APHRC Campus,

Off-Kirawa Road, Manga Cl, Nairobi, Kenya

For peer review only - http://bmjopen.bmj.com/site/about/guidelines.xhtml

Target journal: BMJ open

Abstract

Introduction: Early sexual debut is associated with poor sexual and reproductive health outcomes across the life course. A majority of interventions aimed at delaying sexual debut among adolescents in sub-Saharan Africa (SSA) have been implemented in schools with mixed findings on the effectiveness of such interventions. This systematic review will summarize and synthesize existing evidence on the effectiveness of school-based interventions in delaying sexual debut among adolescents aged 10 – 19 years.

Methods and analysis: We will conduct a comprehensive database search of peer-reviewed studies published in PubMed, Scopus, Science Direct, Web of Science, HINARI and EBSCO (PsycINFO, Global Health, CINAHL), and in Cochrane library, National Institute of Health (NIH), and Turning Research into Practice (TRIP) databases for ongoing studies yet to be published. All studies conducted in SSA between January 2009 and December 2019, regardless of the study design, will be considered. Two authors will independently screen all retrieved records and relevant data on sexual debut extracted.

Data will be pooled using the random effects model. Dichotomous outcomes will be reported as risk ratios and continuous data as mean difference at 95% confidence interval. Heterogeneity will be assessed using the I^2 statistic. Findings will be presented in tables and charts, while providing a description of all included studies, themes and concepts drawn from literature.

Ethics and dissemination: Ethical approval is not required. The findings will be disseminated through peer-reviewed publications, presentations at relevant conferences, and other convening focusing on adolescent sexual and reproductive health.

Strengths and limitations of this study

For peer review only - http://bmjopen.bmj.com/site/about/guidelines.xhtml

Target journal: BMJ open

- This review will synthesise evidence of school-based interventions on delaying sexual debut among adolescents in sub-Saharan Africa.
- The systematic review will adhere to the Preferred Reporting Items for Systematic Reviews and Meta-Analyses (PRISMA) guidelines
- There is a possibility only a small number of diverse studies will be eligible for inclusion in this review, and thus, limited and heterogeneous data for meta-analysis.
- This review will be limited to peer-reviewed studies, published in English between January 2009 and December 2019

For peer review only

Target journal: BMJ open

Background

Adolescents aged 10-19 years in sub-Saharan Africa's (SSA) have a high prevalence of risky sexual behaviours including early sexual debut and unsafe sexual practices (1, 2). Consequently, they are most-at-risk for poor sexual and reproductive health (SRH) outcomes (3-5).

Early sexual debut, described as having had the first sexual intercourse at or before the age of 14 years, increases the period in which an adolescent girl is at risk of getting pregnant (6, 7) and is predominantly driven by individual, familial, contextual and socio-cultural factors (8-10). Early sexual debut is also associated with occurrence of sexual violence, unsafe abortions, unplanned pregnancies, early child marriages, sexually transmitted infections, and HIV infection (11-13), elevated risk of cervical cancer (14, 15) and poor schooling outcomes (11, 16, 17).

Implications of early sexual debut disproportionately affect women and girls, impacting on their health, socio-economic lives and overall wellbeing across the life course (18-26). For instance, young girls who get pregnant at an early age are likely to drop out of school increasing their risk of poor educational and other socio-economic outcomes (27, 28). Young girls are also at greater risk of poor maternal outcomes compared with older women. A review of maternal mortality among adolescents compared with women of other ages in 144 countries revealed a three-fold higher mortality risk among adolescent mothers compared to women above 30 years (2). Similarly, adolescents have higher risk of adverse perinatal outcomes, including low birthweight, preterm delivery, and perinatal death (29-31). The strong association between poor maternal education and poor child-health outcomes such as experiencing severe acute malnutrition, infections as well as poor cognitive growth (32-34), implies that the consequences of early sexual debut transcends generations. Delaying sexual debut is, therefore, a key strategy in averting poor SRH outcomes during and after adolescence.

For peer review only

For peer review only - http://bmjopen.bmj.com/site/about/guidelines.xhtml

Target journal: BMJ open

School is one institution where most adolescents spend most of their time, learning and interacting with peers and adults. Critical thinking developed in schools can be useful for questioning unhealthy behaviours. Schools thus offer a platform for socialization into healthy and unhealthy behaviours (35). As attention turns towards meeting the Sustainable Development Goals (SDGs), the global health community is increasingly committed to adolescent SRH as a prerequisite for improving lives and health of young people (36). This commitment is exemplified by the vast number of diverse school-based interventions targeted at promoting adolescent SRH across the globe, with limited but growing interest in SSA. For instance, schools are being targeted as sites for provision of age-appropriate comprehensive sexuality education that facilitates improved self-efficacy, knowledge and life skills (37-39). Other related school-based interventions such as school fees waivers, supply of menstrual products for girls, school feeding programs for vulnerable populations are also being implemented (40, 41).

However, impact evaluations of school-based interventions on delaying sexual debut suggest mixed findings (40, 42-44). Within high-income countries, the majority of school-based interventions have shown effectiveness in delaying sexual debut (42-44). Similarly, a systematic review and meta-analysis on school based sex education and HIV prevention in low-and middle-income countries globally found that students who received the interventions were less likely to initiate sexual activity (45). In a cluster randomized controlled trial assessing the effects of teacher-led school HIV prevention programs on adolescent sexual risk behaviour in Dar es Salaam, Tanzania and Cape town and Mankweng, South Africa students in Tanzania reported delay in initiating sexual activity during the study while there was no effect of the intervention among students in South Africa (46). Other studies found no significant effects of school-based interventions on delaying sexual debut (47, 48) while others found significant effects (49, 50) found

For peer review only - http://bmjopen.bmj.com/site/about/guidelines.xhtml

Target journal: BMJ open

96 significant effects. These conflicting findings suggests a need for a comprehensive review of school-based interventions to assess their effectiveness on delaying sexual debut.

98 Taken together, these factors underscore the need to synthesize existing studies, and explore the linkages between school-based interventions and early sexual debut in an attempt to inform future adolescent health policies and programing. This systematic review will provide a critical synthesis of existing literature on school-based interventions aimed at delaying early sexual debut among adolescents in SSA, to inform programs, policy and research. The objective of the review is to evaluate the effects of school-based interventions on delaying sexual debut among adolescents in SSA.

105 **Methods and design**

106 This systematic review and meta-analysis will be developed in accordance with the Preferred Reporting Items for Systematic Review and Meta-Analysis Protocols (PRISMA-P) (51). Criteria for considering studies will include study population, type of interventions, type of outcome measures, and type of studies, and study setting, as described in the sections that follow. Important amendments made to this protocol will be documented and published alongside the results of the systematic review.

112 *Study population*

113 The study targets adolescent students aged 10 – 19 years in primary or secondary levels of education or their equivalents who participated in a school-based intervention to delay sexual debut. Studies with students younger than 10 years and/or older than 19 years, as maybe in some settings, will be included if the majority of participants (i.e. above 50%) are aged between 10 and 19 years. Studies that do not include students aged 10 – 19 years will be excluded from the review.

118 *Types of interventions*

For peer review only - http://bmjopen.bmj.com/site/about/guidelines.xhtml

Target journal: BMJ open

All interventions with a school-based component irrespective of intervention content and instruction mode will be considered as long as they assessed sexual debut as a primary or secondary outcome. This includes interventions that are school-based only and delivered in primary and secondary schools, and those that have multiple components, one of which must be a school-based component while the other components are delivered elsewhere (e.g. health care facilities). The intervention must have reported on sexual debut as a primary or secondary outcome. Interventions that target students outside the school setting will be excluded.

Types of outcome

The primary outcome of interest is delayed sexual debut, defined as postponement of sexual intercourse among participants who had not engaged in sexual intercourse prior to the school-based intervention. Secondary outcomes shown in Table 1 will be considered:

Table 1: Secondary outcomes

**Secondary outcomes**	**Definitions**
Intention to delay sex	Planning to wait to have sexual intercourse until older
Lifetime sexual activity	Ever engaged in sexual intercourse
Sexually active	Having engaged in sexual intercourse in the last 30 days
Current sexual activity	Having engaged in sexual intercourse in the last six months
Sexual health knowledge	Knowledge of key SRH topics and issues
Sexual attitude	Attitudes towards sexuality and sexual behaviour
Self-efficacy for safe sex practices	Confidence to say no to unsafe sex practices
Consistent condom use	Condom use at every sexual intercourse

For peer review only - http://bmjopen.bmj.com/site/about/guidelines.xhtml

Target journal: BMJ open

Number of sex-partners	Number of sex partners (regular and casual) in a specified period of time

Types of studies

The review will not be limited to studies using a specific design and meta-analytical approaches that take into consideration different study designs will be used in the analysis. We will also compare the effectiveness of the interventions by study design. Eligible studies will have been published in peer reviewed journals between January 2009 and December 2019. Only studies published in English will be considered.

Study setting

Only studies conducted in SSA will be considered. Given the population of interest, the review will focus on interventions implemented in primary and secondary schools or their equivalents. This review focuses on the period after the global momentum on the need for comprehensive sexuality education began. In 2008, Latin America and the Caribbean signed the Preventing through Education Declaration for the delivery of sexuality education and health services (52). In 2013, there was a Ministerial Commitment on CSE and SRH services for adolescents and young people in 20 countries across Eastern and Southern Africa (53).

Search strategy

Our search strategy will involve three methods:

- Electronic searches - four researchers will search five electronic databases including PubMed, Scopus, Science Direct, Web of Science and EBSCO (PsycINFO, Global Health, CINAHL) for published peer-reviewed journal articles. Cochrane library, National

For peer review only - http://bmjopen.bmj.com/site/about/guidelines.xhtml

Target journal: BMJ open

Institute of Health (NIH), and Turning Research into Practice (TRIP) databases will be searched for ongoing studies that are yet to be published.

- Hand-searches – An iterative process to obtain additional studies not yet retrieved with our initial online database using the reference list of retrieved articles
- Contacting authors and experts - Where published data are not sufficient, authors will be contacted for additional information

While the exact search terms will vary by database, the four search components included will be (1) adolescents (2) sexual debut (3) school-based interventions (4) and sub-Saharan Africa. Search terms will be adapted for each bibliographic database in combination with database-specific filters. Boolean operators 'OR', 'NOT' and 'AND' will be used to maximize or narrow the specificity for the search. Wildcards will be used to search for variations or alternate spellings of key concepts. As an example, we present a draft of search strategy to be used in PubMed below:

1. (intervention[Title/Abstract]) OR (program*[Title/Abstract])
2. (school[Title/Abstract]) OR (institution[Title/Abstract]) OR (academic[Title/Abstract]) OR (education[Title/Abstract])
3. (sexual debut[Title/Abstract]) OR (sexual initiation[Title/Abstract]) OR (sexual delay[Title/Abstract]) OR (sexual activity [Title/Abstract])
4. (adolesce*[Title/Abstract]) OR ("young people"[Title/Abstract]) OR (youth[Title/Abstract]) OR (teenage*[Title/Abstract]) OR (learner[Title/Abstract]) OR (children[Title/Abstract])
5. (Africa south of the Sahara [MeSH Terms]) OR (Africa [MeSH Terms])
6. ("2009/01/01"[Date - Publication]: "2019/12/31"[Date - Publication])

For peer review only - http://bmjopen.bmj.com/site/about/guidelines.xhtml

Target journal: BMJ open

Data collection

Selection of studies

Four reviewers, paired, will independently screen retrieved titles and abstracts of potential articles to determine their eligibility for inclusion in the review. Rayyan, a web application tool will be used to manage the screening process (54). Full article texts of included studies will then be obtained and reviewed to ascertain eligibility. Any disagreements during title and abstract review or during the full text review will be resolved by consensus. A third reviewer will be involved if a consensus is not reached by the two pairs of reviewers.

Data extraction and management

Two reviewers will use a standardized data extraction form to independently extract data on background or process-oriented information from each included study to provide a basis for data charting, themes and variables for use in answering the research question. Reviewers will pilot the data extraction form with a sample of included papers and amendments will be made as necessary. All information will be drawn from the studies highly scored based on the quality of their methodology, robustness of results and level of evidence. From each relevant study, data will be extracted on the following domains:

- General information on the study: authors, date of publication, publication type, country, and funding source
- Study characteristics: study setting, location, study design, sampling frame and sampling methods, and year(s) of study implementation
- Participant characteristics: age, gender, number of participants, participants lost to follow-up, length of follow-up

For peer review only - http://bmjopen.bmj.com/site/about/guidelines.xhtml

Target journal: BMJ open

- Intervention: detailed description of the intervention, composition of intervention and control groups

Outcomes: for primary outcome (delay in sexual debut), data on the number of participants experiencing delayed sexual debut on both intervention versus control arms will be extracted. If a summary estimate (e.g., risk ration) is reported instead of raw numbers, these will be extracted. A similar approach will be used for secondary outcomes. Where more than one article described the same intervention, data will be extracted from all papers. The eligible studies will be exported into the comprehensive meta-analysis (CMA) software.

Risk of bias assessment

Two independent reviewers will assess the methodological quality of studies depending on study design. For RCTs, the reviewers will judge each quality domain based on the following three-point scale as suggested in Cochrane Handbook for Systematic Reviews of Interventions (55): Yes (low risk of bias: plausible bias unlikely to seriously alter the results if all criteria were met); No (high risk of bias: plausible bias that seriously weakens confidence in the results if one or more criteria were not met); and Unclear (plausible bias that raises some doubt about the results if one or more criteria were assessed as unclear. The following items in the risk of bias assessment for RCTs will be included:

- Sequence generation (whether allocation sequence was adequately generated)
- Allocation concealment (whether allocation was adequately concealed)
- Masking/ blinding (whether knowledge of the allocated intervention was adequately prevented during the study, i.e., whether participants, personnel, outcome assessors and/or data analysts are blinded)

For peer review only - http://bmjopen.bmj.com/site/about/guidelines.xhtml

Target journal: BMJ open

- Incomplete outcome reporting (whether incomplete outcome data was adequately addressed)
- Selective reporting (whether reports of the study were free of selective outcome reporting)
- Other sources of data (e.g., whether reports of the study included sample size computation, alpha error, etc.)

For non-RCT studies, the following items will be included in the risk of bias assessment as suggested by Viswanathan, Berkman, Dryden et al. (56):

- Selection bias – do the inclusion/exclusion criteria vary across the comparison groups (for multiple-arm studies) or within groups (for single arm/cross-sectional studies)? Performance bias – does the study fail to account for important variations in the execution of the study from the proposed protocol?
- Detection bias - was the assessor not blinded to the outcome, exposure, or intervention status of the participants? Were valid and reliable measures not used or not implemented consistently across all study participants to assess inclusion/exclusion criteria, intervention/exposure outcomes, participant benefits and harms?
- Attrition bias – was the length of follow-up different across study groups? In cases of missing data was the impact not assessed (e.g., through sensitivity analysis or other adjustment method)?
- Selective outcome reporting - Are any important primary outcomes missing from the results?
- Overall assessment: Are the results believable taking study limitations into consideration?

For peer review only

For peer review only - http://bmjopen.bmj.com/site/about/guidelines.xhtml

Target journal: BMJ open

Discussions and consensus will be used to crosscheck the extracted information and to resolve disagreements. The level of risk of bias in each of these domains will be presented separately for each study in tables in the final review publication.

Risk of bias in individual studies

Methodological quality of each individual article will be appraised using a checklist adapted from Critical Appraisals Skills Programme (CASP). Two authors will independently appraise each article.

Measures of intervention effect

Dichotomous outcomes such as sexual debut (initiated versus not initiated sexual activity during the study period) will be summarized as risk ratios or odds ratio with 95% confidence intervals for each study. Continuous outcomes such as number of sex partners will be summarized as (unstandardized) mean differences and standard errors. In cases where sexual debut may be reported as a median age at sexual debut, published procedures for transforming median to mean and standard deviation to facilitate computation of relevant statistics will be used. If enough information is not provided to calculate an effect size, study authors will be contacted for clarification or to provide additional statistics. If the authors do not provide this information after one month, this study will be removed from the analysis but this effort will be reported.

If results of a repeated measure analysis are reported, authors will need to provide the correlation between pre-post measurements or provide enough information to calculate the correlation between measurements. If these statistics are not available, either in publication or after request, and the study was a controlled design, an effect size will be generated using post-intervention statistics provided groups are similar at baseline with respect to the outcome of interest and other relevant covariates.

For peer review only - http://bmjopen.bmj.com/site/about/guidelines.xhtml

Target journal: BMJ open

Unit of analysis

Data will be extracted from each included study (unit of analysis) as follows. For dichotomous variables, number of participants in the intervention group and the number of participants in the standard/control group will be used. For continuous variables, the mean, standard deviation, and the number of participants in the intervention and control groups will be used. For studies with multiple intervention groups, each pair-wise comparison will be included separately. Moreover, for dichotomous outcomes, we will divide both the number of events and the total number of participants. For continuous outcomes, means and standard deviations will not be changed but will be divided by the total number of participants.

Assessment of heterogeneity

Heterogeneity among studies and between subgroups will be assessed using a Chi² test with a significance level at $p < 0.10$. The degree of heterogeneity among studies and between subgroups will be assessed using the I^2 statistic using the following guide: I^2 = 0% to 40% as heterogeneity that might not be important, I^2 = 30% to 60% as moderate heterogeneity, I^2 = 50% to 90% as substantial heterogeneity, and I^2 = 75% to 100% as considerable heterogeneity (57)

Assessment of reporting bias

Publication bias will be assessed if at least 10 studies are included in the review. A funnel plot will be used to assess the magnitude of reporting bias as per Cochrane guidelines.

Data synthesis

Data will be pooled using the random effects model according to Chapter 9 of the *Cochrane Handbook for Systematic Reviews of Interventions* (57). Dichotomous outcomes' data (e.g., number of participants who experience sexual debut) will be reported as risk ratios and continuous data (e.g., number of sexual partners) as mean differences. All analyses will be done at 95%

For peer review only - http://bmjopen.bmj.com/site/about/guidelines.xhtml

Target journal: BMJ open

confidence intervals. Data will be analysed using the statistical software Comprehensive Meta-Analysis (CMA). In studies where the effects of clustering have not been taken into account, standard deviations will be adjusted for the design effect, using intra-class coefficients, if they are provided in the study reports, or alternatively using external estimates obtained from similar studies. Stratifications will be done by school level (primary or secondary (or age, if applicable), grade level, instructor (e.g., teacher or peer), intervention type (e.g. behaviour change communication, study setting)

Findings will be organised in tables and charts, while presenting a description of the themes and concepts found in literature reflecting the review objectives. A summary narrative that synthesizes the information across tables and charts will be developed, critically highlighting the advances and gaps in research, with a focus to draw implications for future research.

Patient and public involvement

Patients and public will not be involved in the design and conduct of this review

Ethics and dissemination

Ethics approval is not required. The findings of this systematic review will be disseminated through peer-reviewed publications as well as in relevant stakeholders' fora. In case of any amendments to the protocol following its publication, the date of each amendment will be provided, change(s) described, and report the rationale for the change(s) in future publications arising from this protocol.

For peer review only - http://bmjopen.bmj.com/site/about/guidelines.xhtml

Target journal: BMJ open

References

1. Chandra-Mouli V, Camacho AV, Michaud PA. WHO guidelines on preventing early pregnancy and poor reproductive outcomes among adolescents in developing countries. *J Adolesc Health*. 2013;52(5):517-22.
2. Nove A, Matthews Z, Neal S, Camacho AV. Maternal mortality in adolescents compared with women of other ages: Evidence from 144 countries. *Lancet Glob Health*. 2014;2(3):e155-64.
3. Santhya KG, Jejeebhoy SJ. Sexual and reproductive health and rights of adolescent girls: Evidence from low- and middle-income countries. *Glob Public Health*. 2015;10(2):189-221.
4. Decker MR, Peitzmeier S, Olumide A, Acharya R, Ojengbede O, Covarrubias L, et al. Prevalence and health impact of intimate partner violence and non-partner sexual violence among female adolescents aged 15-19 years in vulnerable urban environments: A multi-country study. *J Adolesc Health*. 2014;55(6 Suppl):S58-67.
5. Doyle AM, Mavedzenge SN, Plummer ML, Ross DA. The sexual behaviour of adolescents in sub-Saharan Africa: Patterns and trends from national surveys. *Tropical Medicine & International Health*. 2012;17:796-807.
6. Brahmbhatt H, Kågesten A, Emerson M, Decker MR, Olumide AO, Ojengbede O, et al. Prevalence and determinants of adolescent pregnancy in urban disadvantaged settings across five cities. *J Adolesc Health*. 2014;55(6):S48-S57.
7. O'Donnell L, O'Donnell CR, Stueve A. Early sexual initiation and subsequent sex-related risks among urban minority youth: The reach for health study. *Fam Plann Perspect*. 2001:268-75.

For peer review only - http://bmjopen.bmj.com/site/about/guidelines.xhtml

Target journal: BMJ open

8. Ahorlu CK, Pfeiffer C, Obrist B. Socio-cultural and economic factors influencing adolescents' resilience against the threat of teenage pregnancy: A cross-sectional survey in Accra, Ghana. *Reprod Health*. 2015;12:117.

9. Ajayi AI, Okeke SR. Protective sexual behaviours among young adults in Nigeria: Influence of family support and living with both parents. *BMC Public Health*. 2019;19(1):983.

10. Bingenheimer JB, Asante E, Ahiadeke C. Peer influences on sexual activity among adolescents in Ghana. *Stud Fam Plann*. 2015;46(1):1-19.

11. Durowade KA, Babatunde OA, Omokanye LO, Elegbede OE, Ayodele LM, Adewoye KR, et al. Early sexual debut: prevalence and risk factors among secondary school students in Ido-ekiti, Ekiti state, South-West Nigeria. *Afr Health Sci*. 2017;17(3):614-22.

12. Makenzius M, Larsson M. Early onset of sexual intercourse is an indicator for hazardous lifestyle and problematic life situation. *Scand J Caring Sci*. 2013;27(1):20-6.

13. Peltzer K. Early sexual debut and associated factors among in-school adolescents in eight African countries. *Acta paediatrica (Oslo, Norway : 1992)*. 2010;99(8):1242-7.

14. Louie KS, de Sanjose S, Diaz M, Castellsagué X, Herrero R, Meijer CJ, et al. Early age at first sexual intercourse and early pregnancy are risk factors for cervical cancer in developing countries. *Br J Cancer*. 2009;100(7):1191-7.

15. Plummer M, Peto J, Franceschi S. Time since first sexual intercourse and the risk of cervical cancer. *Int J Cancer*. 2012;130(11):2638-44.

16. Kirby D. The impact of schools and school programs upon adolescent sexual behavior. *J Sex Res*. 2002;39(1):27-33.

For peer review only

For peer review only - http://bmjopen.bmj.com/site/about/guidelines.xhtml

Target journal: BMJ open

17. Bay-Cheng LY. The trouble of teen sex: The construction of adolescent sexuality through school-based sexuality education. *Sex Educ*. 2003;3(1):61-74.
18. Agardh A, Cantor-Graae E, Östergren P-O. Youth, sexual risk-taking behavior, and mental health: A study of university students in Uganda. *Int J Behav Med*. 2012;19(2):208-16.
19. Boislard M-AP, Dussault F, Brendgen M, Vitaro F. Internalizing and externalizing behaviors as predictors of sexual onset in early adolescence. *J Early Adolesc*. 2013.
20. Brown LK, Hadley W, Stewart A, Lescano C, Whiteley L, Donenberg G, et al. Psychiatric disorders and sexual risk among adolescents in mental health treatment. *J Consult Clin Psychol*. 2010;78(4):590-7.
21. Callahan MR, Tolman RM, Saunders DG. Adolescent dating violence victimization and psychological well-being. *J Adolesc Res*. 2003;18(6):664-81.
22. Kabiru CW, Izugbara CO, Beguy D. The health and wellbeing of young people in sub-Saharan Africa: An under-researched area? *BMC Int Health Hum Rights*. 2013;13(1):11.
23. Kleinert S, Horton R. Adolescent health and wellbeing: A key to a sustainable future. *Lancet*. 2016;387(10036):2355-6.
24. Patton GC, Sawyer SM, Santelli JS, Ross DA, Afifi R, Allen NB, et al. Our future: A Lancet commission on adolescent health and wellbeing. *The Lancet*. 2016;387(10036):2423-78.
25. Rock A, Barrington C, Abdoulayi S, Tsoka M, Mvula P, Handa S. Social networks, social participation, and health among youth living in extreme poverty in rural Malawi. *Soc Sci Med*. 2016;170:55-62.
26. Viner RM, Ozer EM, Denny S, Marmot M, Resnick M, Fatusi A, et al. Adolescence and the social determinants of health. *Lancet*. 2012;379:1641-52.

For peer review only

For peer review only - http://bmjopen.bmj.com/site/about/guidelines.xhtml

Target journal: BMJ open

27. Beguy D, Ndugwa R, Kabiru CW. Entry into motherhood among adolescent girls in two informal settlements in Nairobi, Kenya. *J Biosoc Sci*. 2013;45(6):721-42.

28. Gonçalves H, Machado EC, Soares ALG, Camargo-Figuera FA, Seering LM, Mesenburg MA, et al. Sexual initiation among adolescents (10 to 14 years old) and health behaviors. *Rev Bras Epidemiol*. 2015;18:25-41.

29. Althabe F, Moore JL, Gibbons L, Berrueta M, Goudar SS, Chomba E, et al. Adverse maternal and perinatal outcomes in adolescent pregnancies: The Global Network's Maternal Newborn Health Registry study. *Reprod Health*. 2015;12 Suppl 2(Suppl 2):S8.

30. Moraes AN, Likwa RN, Nzala SH. A retrospective analysis of adverse obstetric and perinatal outcomes in adolescent pregnancy: The case of Luapula Province, Zambia. *Matern Health Neonatol Perinatol*. 2018;4(1):20.

31. Marvin-Dowle K, Kilner K, Burley VJ, Soltani H. Impact of adolescent age on maternal and neonatal outcomes in the Born in Bradford cohort. *BMJ Open*. 2018;8(3):e016258.

32. Güneş P. The role of maternal education in child health: Evidence from a compulsory schooling law. *Econ Educ Rev*. 2015;47.

33. Mensch BS, Chuang EK, Melnikas AJ, Psaki SR. Evidence for causal links between education and maternal and child health: Systematic review. *Trop Med Int Health*. 2019;24(5):504-22.

34. Prickett KC, Augustine JM. Maternal education and investments in children's health. *J Marriage Fam*. 2016;78(1):7-25.

35. WHO. *Promoting health through schools. Global school health initiative*. WHO, 1996.

36. WHO. *Global Accelerated Action for the Health of Adolescents (AA-HA!): Guidance to support country implementation*. Geneva: World Health Organization; 2017.

For peer review only - http://bmjopen.bmj.com/site/about/guidelines.xhtml

Target journal: BMJ open

395 37. Allensworth DD, Kolbe LJ. The Comprehensive School Health Program: Exploring an expanded concept. *J Sch Health*. 1987;57(10):409-12.

397 38. Chirwa-Kambole E, Svanemyr J, Sandøy I, Hangoma P, Zulu JM. Acceptability of youth clubs focusing on comprehensive sexual and reproductive health education in rural Zambian schools: A case of Central Province. *BMC Health Serv Res*. 2020;20(1):42.

400 39. Deschesnes M, Martin C, Hill AJ. Comprehensive approaches to school health promotion: How to achieve broader implementation? *Health Promot Int*. 2003;18(4):387-96.

402 40. Mason-Jones AJ, Sinclair D, Mathews C, Kagee A, Hillman A, Lombard C. School-based interventions for preventing HIV, sexually transmitted infections, and pregnancy in adolescents. *Cochrane Database Syst Rev*. 2016(11).

405 41. Salam RA, Faqqah A, Sajjad N, Lassi ZS, Das JK, Kaufman M, et al. Improving adolescent sexual and reproductive health: A systematic review of potential interventions. *J Adolesc Health*. 2016;59(4):S11-S28.

408 42. Denford S, Abraham C, Campbell R, Busse H. A comprehensive review of reviews of school-based interventions to improve sexual-health. *Health Psychol Rev*. 2017;11(1):33-52.

411 43. Wang T, Lurie M, Govindasamy D, Mathews C. The effects of school-based condom availability programs (CAPs) on condom acquisition, use and sexual behavior: A systematic review. *AIDS Behav*. 2018;22(1):308-20.

414 44. Koo HP, Rose A, El-Khorazaty MN, Yao Q, Jenkins RR, Anderson KM, et al. Evaluation of a randomized intervention to delay sexual initiation among fifth-graders followed through the sixth grade. *Sex Educ*. 2011;11(01):27-46.

For peer review only - http://bmjopen.bmj.com/site/about/guidelines.xhtml

Target journal: BMJ open

45. Fonner VA, Armstrong KS, Kennedy CE, O'Reilly KR, Sweat MD. School based sex education and HIV prevention in low- and middle-income countries: A systematic review and meta-analysis. *PLoS One*. 2014;9(3):e89692-e.

46. Mathews C, Aarø LE, Grimsrud A, Flisher AJ, Kaaya S, Onya H, et al. Effects of the SATZ teacher-led school HIV prevention programmes on adolescent sexual behaviour: Cluster randomised controlled trials in three sub-Saharan African sites. *Int Health*. 2012;4(2):111-22.

47. Jemmott JB, Jemmott LS, O'Leary A, Ngwane Z, Icard LD, Bellamy SL, et al. School-based randomized controlled trial of an HIV/STD risk-reduction intervention for South African adolescents. *Arch Pediatr Adolesc Med*. 2010;164(10):923-9.

48. Cowan FM, Pascoe SJ, Langhaug LF, Mavhu W, Chidiya S, Jaffar S, et al. The Regai Dzive Shiri Project: Results of a randomised trial of an HIV prevention intervention for Zimbabwean youth. *AIDS*. 2010;24(16):2541.

49. Maticka-Tyndale E, Wildish J, Gichuru M. Thirty-month quasi-experimental evaluation follow-up of a national primary school HIV intervention in Kenya. *Sex Educ*. 2010;10(2):113-30.

50. Mmbaga EJ, Kajula L, Aarø LE, Kilonzo M, Wubs AG, Eggers SM, et al. Effect of the PREPARE intervention on sexual initiation and condom use among adolescents aged 12–14: A cluster randomised controlled trial in Dar es Salaam, Tanzania. *BMC Public Health*. 2017;17(1):322.

51. Moher D, Shamseer L, Clarke M, Ghersi D, Liberati A, Petticrew M, et al. Preferred reporting items for systematic review and meta-analysis protocols (PRISMA-P) 2015 statement. *Systematic reviews*. 2015;4(1):1.

For peer review only - http://bmjopen.bmj.com/site/about/guidelines.xhtml

Target journal: BMJ open

52. Declaration M, editor Preventing through education. First meeting of Ministers of Health and Education to Stop HIV and STIs in Latin America and the Caribbean (Mexico City: 1 August 2008); 2008.
53. Ministries of Education, Ministries of Health. *Ministerial commitment on comprehensive sexuality education and sexual and reproductive health services for adolescents and young people in Eastern and Southern Africa (ESA)*. Angola, Botswana, Burundi, Democratic Republic of Congo, Ethiopia, Kenya…; 2013.
54. Ouzzani M, Hammady H, Fedorowicz Z, Elmagarmid A. *Rayyan — a web and mobile app for systematic reviews*. Systematic Reviews; 2016.
55. Higgins JP, Thomas J, Chandler J, Cumpston M, Li T, Page MJ, et al. *Cochrane handbook for systematic reviews of interventions*: John Wiley & Sons; 2019.
56. Viswanathan M, Berkman ND, Dryden DM, Hartling L. *Assessing risk of bias and confounding in observational studies of interventions or exposures: further development of the RTI item bank*. RTI International–University of North Carolina at Chapel Hill Evidence-based Practice Center, Research Triangle Park, NC; The University of Alberta Evidence-based Practice Center, Edmonton, Canada, 2013.
57. Deeks JJ, Higgins JP, Altman DG, Group CSM. *Analysing data and undertaking meta‐analyses*: John Wiley & Sons; 2019. 241-84 p.

For peer review only - http://bmjopen.bmj.com/site/about/guidelines.xhtml

Target journal: BMJ open

Author contribution: BWM, KJ, EKI and JO contributed to the conceptualization, and development of the protocol. BWM and KJ provided the initial draft of the protocol. HW and CWK provided a critical review of protocol. All authors read and approved the final draft of the protocol.

Funding statement: Authors' time to develop this protocol was supported by the International Development Research Centre (Grant number 108676-002) and the Swedish International Development Cooperation Agency (Grant number 12103)

Competing interests: None declared

Patients consent for publication: Not required

Word Count: 3,002 words (excludes the title page, abstract, tables, acknowledgements, contributions and references

For peer review only

PRISMA-P 2015 Checklist

This checklist has been adapted for use with protocol submissions to *Systematic Reviews* from Table 3 in Moher D et al: Preferred reporting items for systematic review and meta-analysis protocols (PRISMA-P) 2015 statement. *Systematic Reviews* 2015 **4**:1

Section/topic	#	Checklist item	Information reported		Line number(s)
			Yes	No	
**ADMINISTRATIVE INFORMATION**					
**Title**					
Identification	1a	Identify the report as a protocol of a systematic review	☒	☐	1 - 2
Update	1b	If the protocol is for an update of a previous systematic review, identify as such	☐	☒	Not applicable
**Registration**	2	If registered, provide the name of the registry (e.g., PROSPERO) and registration number in the Abstract	☐	☒	Not registered
**Authors**					
Contact	3a	Provide name, institutional affiliation, and e-mail address of all protocol authors; provide physical mailing address of corresponding author	☒	☐	6 - 15
Contributions	3b	Describe contributions of protocol authors and identify the guarantor of the review	☒	☐	447 - 450
**Amendments**	4	If the protocol represents an amendment of a previously completed or published protocol, identify as such and list changes; otherwise, state plan for documenting important protocol amendments	☒	☐	98 - 100
**Support**					
Sources	5a	Indicate sources of financial or other support for the review	☒	☐	451 - 452
Sponsor	5b	Provide name for the review funder and/or sponsor	☒	☐	451 - 452
Role of sponsor/funder	5c	Describe roles of funder(s), sponsor(s), and/or institution(s), if any, in developing the protocol	☒	☐	451 - 452
**INTRODUCTION**					
**Rationale**	6	Describe the rationale for the review in the context of what is already known	☒	☐	74 - 91
**Objectives**	7	Provide an explicit statement of the question(s) the review will address with reference to participants, interventions, comparators, and outcomes (PICO)	☒	☐	91 - 93

BioMed Central The Open Access Publisher

For peer review only - http://bmjopen.bmj.com/site/about/guidelines.xhtml

Section/topic	#	Checklist item	Information reported Yes	Information reported No	Line number(s)
**METHODS**					
**Eligibility criteria**	8	Specify the study characteristics (e.g., PICO, study design, setting, time frame) and report characteristics (e.g., years considered, language, publication status) to be used as criteria for eligibility for the review	☒	☐	95 - 134
**Information sources**	9	Describe all intended information sources (e.g., electronic databases, contact with study authors, trial registers, or other grey literature sources) with planned dates of coverage	☒	☐	135 - 151
**Search strategy**	10	Present draft of search strategy to be used for at least one electronic database, including planned limits, such that it could be repeated	☒	☐	152 - 161
STUDY RECORDS					
Data management	11a	Describe the mechanism(s) that will be used to manage records and data throughout the review	☒	☐	164 - 192
Selection process	11b	State the process that will be used for selecting studies (e.g., two independent reviewers) through each phase of the review (i.e., screening, eligibility, and inclusion in meta-analysis)	☒	☐	164 - 170
Data collection process	11c	Describe planned method of extracting data from reports (e.g., piloting forms, done independently, in duplicate), any processes for obtaining and confirming data from investigators	☒	☐	172 - 177
**Data items**	12	List and define all variables for which data will be sought (e.g., PICO items, funding sources), any pre-planned data assumptions and simplifications	☒	☐	177 - 192
**Outcomes and prioritization**	13	List and define all outcomes for which data will be sought, including prioritization of main and additional outcomes, with rationale	☒	☐	115 - 119
**Risk of bias in individual studies**	14	Describe anticipated methods for assessing risk of bias of individual studies, including whether this will be done at the outcome or study level, or both; state how this information will be used in data synthesis	☒	☐	193- 230
DATA					
**Synthesis**	15a	Describe criteria under which study data will be quantitatively synthesized	☒	☐	270 - 285
	15b	If data are appropriate for quantitative synthesis, describe planned summary measures, methods of handling data, and methods of combining data from studies, including any planned exploration of consistency (e.g., I^2, Kendall's tau)	☒	☐	235 - 265
	15c	Describe any proposed additional analyses (e.g., sensitivity or subgroup analyses, meta-regression)	☒	☐	275 – 277
	15d	If quantitative synthesis is not appropriate, describe the type of summary planned	☒	☐	282 - 285
**Meta-bias(es)**	16	Specify any planned assessment of meta-bias(es) (e.g., publication bias across studies, selective reporting within studies)	☒	☐	276 - 281
**Confidence in cumulative evidence**	17	Describe how the strength of the body of evidence will be assessed (e.g., GRADE)	☒	☐	231 - 234

For peer review only

For peer review only - http://bmjopen.bmj.com/site/about/guidelines.xhtml

3

For peer review only

BioMed Central
The Open Access Publisher

BMJ Open

Effectiveness of school-based interventions in delaying sexual debut among adolescents in sub-Saharan Africa: A protocol for a systematic review and meta-analysis

Journal:	*BMJ Open*
Manuscript ID	bmjopen-2020-044398.R1
Article Type:	Protocol
Date Submitted by the Author:	12-Mar-2021
Complete List of Authors:	Maina, Beatrice; African Population and Health Research Center, Population Dynamics and Reproductive Health; University of the Witwatersrand Faculty of Health Sciences, School of Public Health<br>Juma, Kenneth ; African Population and Health Research Center, Population Dynamics and Sexual Reproductive Health<br>Igonya, Emmy; African Population and Health Research Center, PDRH<br>Osindo, Jane; African Population and Health Research Center<br>Wao, Hesborn; African Population and Health Research Center<br>Kabiru, Caroline; African Population and Health Research Center
**Primary Subject Heading**:	Public health
Secondary Subject Heading:	Sexual health, Global health
Keywords:	PUBLIC HEALTH, Health policy < HEALTH SERVICES ADMINISTRATION & MANAGEMENT, Community child health < PAEDIATRICS

SCHOLARONE™
Manuscripts

I, the Submitting Author has the right to grant and does grant on behalf of all authors of the Work (as defined in the below author licence), an exclusive licence and/or a non-exclusive licence for contributions from authors who are: i) UK Crown employees; ii) where BMJ has agreed a CC-BY licence shall apply, and/or iii) in accordance with the terms applicable for US Federal Government officers or employees acting as part of their official duties; on a worldwide, perpetual, irrevocable, royalty-free basis to BMJ Publishing Group Ltd ("BMJ") its licensees and where the relevant Journal is co-owned by BMJ to the co-owners of the Journal, to publish the Work in this journal and any other BMJ products and to exploit all rights, as set out in our licence.

The Submitting Author accepts and understands that any supply made under these terms is made by BMJ to the Submitting Author unless you are acting as an employee on behalf of your employer or a postgraduate student of an affiliated institution which is paying any applicable article publishing charge ("APC") for Open Access articles. Where the Submitting Author wishes to make the Work available on an Open Access basis (and intends to pay the relevant APC), the terms of reuse of such Open Access shall be governed by a Creative Commons licence – details of these licences and which Creative Commons licence will apply to this Work are set out in our licence referred to above.

Other than as permitted in any relevant BMJ Author's Self Archiving Policies, I confirm this Work has not been accepted for publication elsewhere, is not being considered for publication elsewhere and does not duplicate material already published. I confirm all authors consent to publication of this Work and authorise the granting of this licence.

For peer review only

Effectiveness of school-based interventions in delaying sexual debut among adolescents in sub-Saharan Africa: A protocol for a systematic review and meta-analysis

Short Title: School-based interventions and sexual debut

Authors: Beatrice W. Maina[1], Kenneth Juma[1], Emmy Kageha Igonya[1], Jane Osindo[1], Hesborn Wao[1], Caroline W. Kabiru[1]

Affiliation

[1]African Population Health and Research Center, Nairobi, Kenya

Corresponding Author

Beatrice W. Maina

Email: bmaina@aphrc.org

P.O. Box 10787, APHRC Campus,

Off-Kirawa Road, Manga Cl, Nairobi, Kenya

For peer review only - http://bmjopen.bmj.com/site/about/guidelines.xhtml

Abstract

Introduction: Early sexual debut is associated with poor sexual and reproductive health outcomes across the life course. A majority of interventions aimed at delaying sexual debut among adolescents in sub-Saharan Africa (SSA) have been implemented in schools with mixed findings on the effectiveness of such interventions. This systematic review will summarize and synthesize existing evidence on the effectiveness of school-based interventions in delaying sexual debut among adolescents aged 10 – 19 years.

Methods and analysis: We will conduct a comprehensive database search of peer-reviewed studies published in PubMed, Scopus, Science Direct, Web of Science, HINARI and EBSCO (PsycINFO, Global Health, CINAHL), and in Cochrane library, National Institute of Health (NIH), and Turning Research into Practice (TRIP) databases for ongoing studies yet to be published. All studies conducted in SSA between January 2009 and December 2020, regardless of the study design, will be included. Two authors will independently screen all retrieved records and relevant data on sexual debut extracted.

Data will be pooled using the random effects model. Dichotomous outcomes will be reported as risk ratios and continuous data as mean difference at 95% confidence interval. Heterogeneity will be assessed using the I^2 statistic. Findings will be presented in tables and charts, while providing a description of all included studies, themes and concepts drawn from literature.

Ethics and dissemination: Ethical approval is not required. The findings will be disseminated through peer-reviewed publications, presentations at relevant conferences, and other convening focusing on adolescent sexual and reproductive health.

Strengths and limitations of this study

For peer review only - http://bmjopen.bmj.com/site/about/guidelines.xhtml

- To the best of our knowledge, this is the first systematic review and meta-analysis to focus on the effectiveness of school-based interventions on delaying sexual debut among adolescents in sub-Saharan Africa.
- The systematic review and meta-analysis includes all interventions with a school-based component regardless of the study design used in the study.
- The systematic review and meta-analysis will adhere to the Preferred Reporting Items for Systematic Reviews and Meta Analyses (PRISMA) guidelines.
- To minimise the likelihood of reviewer bias, two reviewers will screen for study eligibility and perform the quality assessment.
- This review will be limited to peer-reviewed studies, published in English between January 2009 and December 2020

For peer review only

Background

Adolescents aged 10-19 years in sub-Saharan Africa's (SSA) have a high prevalence of risky sexual behaviours including early sexual debut and unsafe sexual practices (1, 2). Consequently, they are most-at-risk for poor sexual and reproductive health (SRH) outcomes (3-5).

Early sexual debut, described as having had the first sexual intercourse at or before the age of 14 years, increases the period in which an adolescent girl is at risk of getting pregnant (6, 7) and is predominantly driven by individual, familial, contextual and socio-cultural factors (8-10). Early sexual debut is also associated with occurrence of sexual violence, unsafe abortions, unplanned pregnancies, early child marriages, sexually transmitted infections, and HIV infection (11-13), elevated risk of cervical cancer (14, 15) and poor schooling outcomes (11, 16, 17).

Implications of early sexual debut disproportionately affect women and girls, impacting on their health, socio-economic lives and overall wellbeing across the life course (18-26). For instance, young girls who get pregnant at an early age are likely to drop out of school increasing their risk of poor educational and other socio-economic outcomes (27, 28). Young girls are also at greater risk of poor maternal outcomes compared with older women. A review of maternal mortality among adolescents compared with women of other ages in 144 countries revealed a three-fold higher mortality risk among adolescent mothers compared to women above 30 years (2). Similarly, adolescents have higher risk of adverse perinatal outcomes, including low birthweight, preterm delivery, and perinatal death (29-31). The strong association between poor maternal education and poor child-health outcomes such as experiencing severe acute malnutrition, infections as well as poor cognitive growth (32-34), implies that the consequences of early sexual debut transcends generations. Delaying sexual debut is, therefore, a key strategy in averting poor SRH outcomes during and after adolescence.

For peer review only - http://bmjopen.bmj.com/site/about/guidelines.xhtml

School is one institution where most adolescents spend most of their time, learning and interacting with peers and adults. Critical thinking developed in schools can be useful for questioning unhealthy behaviours. Schools thus offer a platform for socialization into healthy and unhealthy behaviours (35). As attention turns towards meeting the Sustainable Development Goals (SDGs), the global health community is increasingly committed to adolescent SRH as a prerequisite for improving lives and health of young people (36). This commitment is exemplified by the vast number of diverse school-based interventions targeted at promoting adolescent SRH across the globe, with limited but growing interest in SSA. For instance, schools are being targeted as sites for provision of age-appropriate comprehensive sexuality education that facilitates improved self-efficacy, knowledge and life skills (37-39). Other related school-based interventions such as school fees waivers, supply of menstrual products for girls, school feeding programs for vulnerable populations are also being implemented (40, 41).

However, impact evaluations of school-based interventions on delaying sexual debut suggest mixed findings (40, 42-44). Within high-income countries, the majority of school-based interventions have shown effectiveness in delaying sexual debut (42-44). Similarly, a systematic review and meta-analysis on school based sex education and HIV prevention in low-and middle-income countries globally found that students who received the interventions were less likely to initiate sexual activity (45). In a cluster randomized controlled trial assessing the effects of teacher-led school HIV prevention programs on adolescent sexual risk behaviour in Dar es Salaam, Tanzania and Cape town and Mankweng, South Africa students in Tanzania reported delay in initiating sexual activity during the study while there was no effect of the intervention among students in South Africa (46). Other studies found no significant effects of school-based interventions on delaying sexual debut (47, 48) while others found significant effects (49, 50) found

For peer review only - http://bmjopen.bmj.com/site/about/guidelines.xhtml

significant effects. These conflicting findings suggests a need for a comprehensive review of school-based interventions to assess their effectiveness on delaying sexual debut.

Taken together, these factors underscore the need to synthesize existing studies, and explore the linkages between school-based interventions and early sexual debut in an attempt to inform future adolescent health policies and programing. This systematic review will provide a critical synthesis of existing literature on school-based interventions aimed at delaying early sexual debut among adolescents in SSA, to inform programs, policy and research. The objective of the review is to evaluate the effects of school-based interventions on delaying sexual debut among adolescents in SSA.

Methods and design

This systematic review and meta-analysis will be developed in accordance with the Preferred Reporting Items for Systematic Review and Meta-Analysis Protocols (PRISMA-P) (51). Criteria for considering studies will include study population, type of interventions, type of outcome measures, and type of studies, and study setting, as described in the sections that follow. Important amendments made to this protocol will be documented and published alongside the results of the systematic review.

Study population

The study targets adolescent students aged 10 – 19 years in primary or secondary levels of education or their equivalents who participated in a school-based intervention to delay sexual debut. Studies with students younger than 10 years and/or older than 19 years, as maybe in some settings, will be included if the majority of participants (i.e. above 50%) are aged between 10 and 19 years. Studies that do not include students aged 10 – 19 years will be excluded from the review.

Types of interventions

For peer review only - http://bmjopen.bmj.com/site/about/guidelines.xhtml

122 All interventions with a school-based component irrespective of intervention content and instruction mode will be included as long as they assessed sexual debut as a primary or secondary outcome. This includes interventions that are school-based only and delivered in primary and secondary schools, and those that have multiple components, one of which must be a school-based component while the other components are delivered elsewhere (e.g. health care facilities). The intervention must have reported on sexual debut as a primary or secondary outcome. Interventions that target students outside the school setting will be excluded.

Types of outcome

The primary outcome of interest is delayed sexual debut, defined as postponement of sexual intercourse among participants who had not engaged in sexual intercourse prior to the school-based intervention. While secondary outcomes shown in Table 1 will be considered as a first step, other SRH outcomes reported by at least two studies will also be considered. Such may include contraceptive use, pregnancy and history of STIs:

Table 1: Secondary outcomes

**Secondary outcomes**	**Definitions**
Intention to delay sex	Planning to wait to have sexual intercourse until older
Lifetime sexual activity	Ever engaged in sexual intercourse
Sexually active	Having engaged in sexual intercourse in the last 30 days
Current sexual activity	Having engaged in sexual intercourse in the last six months
Sexual health knowledge	Knowledge of key SRH topics and issues
Sexual attitude	Attitudes towards sexuality and sexual behaviour
Self-efficacy for safe sex practices	Confidence to say no to unsafe sex practices

For peer review only - http://bmjopen.bmj.com/site/about/guidelines.xhtml

Consistent condom use	Condom use at every sexual intercourse
Number of sex-partners	Number of sex partners (regular and casual) in a specified period of time

136

137 *Types of studies*

138 The review will include all studies focused on delaying sexual debut, regardless of the study design
139 employed. Meta-analytical approaches that take into consideration different study designs will
140 be used in the analysis, and where meta-analysis is not possible, other methods of analysing the
141 effect measures outlined by McKenzie and Brennan (52) will be employed . We will also compare
142 the effectiveness of the interventions by study design. Eligible studies will have been published in
143 peer reviewed journals between January 2009 and December 2020. Only studies published in
144 English will be included.

145 *Study setting*

146 Only studies conducted in SSA will be included. Given the population of interest, the review will
147 focus on interventions implemented in primary and secondary schools or their equivalents. The
148 review focuses on studies published since 2009, the period after the global momentum on the need
149 for comprehensive sexuality education began. In 2008, Latin America and the Caribbean signed
150 the Preventing through Education Declaration for the delivery of sexuality education and health
151 services (53). In 2013, there was a Ministerial Commitment on CSE and SRH services for
152 adolescents and young people in 20 countries across Eastern and Southern Africa (54).

153 *Search strategy*

154 Our search strategy will involve three methods:

For peer review only - http://bmjopen.bmj.com/site/about/guidelines.xhtml

- Electronic searches - four researchers will search five electronic databases including PubMed, Scopus, Science Direct, Web of Science and EBSCO (PsycINFO, Global Health, CINAHL) for published peer-reviewed journal articles. Cochrane library, National Institute of Health (NIH), and Turning Research into Practice (TRIP) databases will be searched for ongoing studies that are yet to be published.
- Hand-searches – An iterative process to obtain additional studies not yet retrieved with our initial online database using the reference list of retrieved articles
- Contacting authors and experts - Where published data are not sufficient, authors will be contacted for additional information

While the exact search terms will vary by database, the four search components included will be (1) adolescents (2) sexual debut (3) school-based interventions (4) and sub-Saharan Africa. Search terms will be adapted for each bibliographic database in combination with database-specific filters. Boolean operators 'OR', 'NOT' and 'AND' will be used to maximize or narrow the specificity for the search. Wildcards will be used to search for variations or alternate spellings of key concepts. Below, and in the supplementary document, we present a search strategy to be used in PubMed below:

1. (intervention[Title/Abstract]) OR (program*[Title/Abstract])
2. (school[Title/Abstract]) OR (institution[Title/Abstract]) OR (academic[Title/Abstract]) OR (education[Title/Abstract])
3. (sexual debut[Title/Abstract]) OR (sexual initiation[Title/Abstract]) OR (sexual delay[Title/Abstract]) OR (sexual activity [Title/Abstract])

For peer review only

For peer review only - http://bmjopen.bmj.com/site/about/guidelines.xhtml

4. (adolesce*[Title/Abstract]) OR ("young people"[Title/Abstract]) OR (youth[Title/Abstract]) OR (teenage*[Title/Abstract]) OR (learner[Title/Abstract]) OR (children[Title/Abstract])
5. (Africa south of the Sahara [MeSH Terms]) OR (Africa [MeSH Terms])
6. ("2009/01/01"[Date - Publication]: "2020/12/31"[Date - Publication])
7. #1 AND #2 AND #3 AND #4 AND #5 AND #6

("intervention"[Title/Abstract] OR "program*"[Title/Abstract]) AND ("school"[Title/Abstract] OR "institution"[Title/Abstract] OR "academic"[Title/Abstract] OR "education"[Title/Abstract]) AND ("sexual debut"[Title/Abstract] OR "sexual initiation"[Title/Abstract] OR "sexual delay"[Title/Abstract] OR "sexual activity"[Title/Abstract]) AND ("adolesce*"[Title/Abstract] OR "young people"[Title/Abstract] OR "youth"[Title/Abstract] OR "teenage*"[Title/Abstract] OR "learner"[Title/Abstract] OR "children"[Title/Abstract]) AND ("africa south of the sahara"[MeSH Terms] OR "africa"[MeSH Terms]) AND 2009/01/01:2020/12/31[Date - Publication]

For peer review only

Data collection

Selection of studies

Four reviewers, paired, will independently screen retrieved titles and abstracts of potential articles to determine their eligibility for inclusion in the review. Rayyan, a web application tool will be used to manage the screening process (55). Full article texts of included studies will then be obtained and reviewed to ascertain eligibility. Any disagreements during title and abstract review

For peer review only - http://bmjopen.bmj.com/site/about/guidelines.xhtml

or during the full text review will be resolved by consensus. A third reviewer will be involved if a consensus is not reached by the two pairs of reviewers.

Data extraction and management

Two reviewers will use a standardized data extraction form to independently extract data on background or process-oriented information from each included study to provide a basis for data charting, themes and variables for use in answering the research question. Reviewers will pilot the data extraction form with a sample of included papers and amendments will be made as necessary. From each relevant study, data will be extracted on the following domains:

- General information on the study: authors, date of publication, publication type, country, and funding source
- Study characteristics: study setting, location, study design, sampling frame and sampling methods, and year(s) of study implementation
- Participant characteristics: age, gender, number of participants, participants lost to follow-up, length of follow-up
- Intervention: detailed description of the intervention, composition of intervention and control groups

Outcomes: for primary outcome (delay in sexual debut), data on the number of participants experiencing delayed sexual debut on both intervention versus control arms will be extracted. If a summary estimate (e.g., risk ration) is reported instead of raw numbers, these will be extracted. A similar approach will be used for secondary outcomes. Where more than one article described the same intervention, data will be extracted from all papers. The eligible studies will be exported into the Comprehensive Meta-Analysis (56)software.

Risk of bias assessment

For peer review only - http://bmjopen.bmj.com/site/about/guidelines.xhtml

221 Two independent reviewers will assess the methodological quality of studies depending on study design. For RCTs, the reviewers will judge each quality domain based on the following three-point scale as suggested in Cochrane Handbook for Systematic Reviews of Interventions (57): Yes (low risk of bias: plausible bias unlikely to seriously alter the results if all criteria were met); No (high risk of bias: plausible bias that seriously weakens confidence in the results if one or more criteria were not met); and Unclear (plausible bias that raises some doubt about the results if one or more criteria were assessed as unclear. The following items in the risk of bias assessment for RCTs will be included:

- Sequence generation (whether allocation sequence was adequately generated)
- Allocation concealment (whether allocation was adequately concealed)
- Masking/ blinding (whether knowledge of the allocated intervention was adequately prevented during the study, i.e., whether participants, personnel, outcome assessors and/or data analysts are blinded)
- Incomplete outcome reporting (whether incomplete outcome data was adequately addressed)
- Selective reporting (whether reports of the study were free of selective outcome reporting)
- Other sources of data (e.g., whether reports of the study included sample size computation, alpha error, etc.)

For non-RCT studies, the following items will be included in the risk of bias assessment as suggested by Viswanathan, Berkman, Dryden et al. (58):

- Selection bias – do the inclusion/exclusion criteria vary across the comparison groups (for multiple-arm studies) or within groups (for single arm/cross-sectional studies)?

For peer review only

Performance bias – does the study fail to account for important variations in the execution of the study from the proposed protocol?

- Detection bias - was the assessor not blinded to the outcome, exposure, or intervention status of the participants? Were valid and reliable measures not used or not implemented consistently across all study participants to assess inclusion/exclusion criteria, intervention/exposure outcomes, participant benefits and harms?
- Attrition bias – was the length of follow-up different across study groups? In cases of missing data was the impact not assessed (e.g., through sensitivity analysis or other adjustment method)?
- Selective outcome reporting - Are any important primary outcomes missing from the results?
- Overall assessment: Are the results believable taking study limitations into consideration?

Discussions and consensus will be used to crosscheck the extracted information and to resolve disagreements. The level of risk of bias in each of these domains will be presented separately for each study in tables in the final review publication.

Risk of bias in individual studies

Methodological quality of each individual article will be appraised using a checklist adapted from Critical Appraisals Skills Programme (CASP). Two authors will independently appraise each article.

Measures of intervention effect

Dichotomous outcomes such as sexual debut (initiated versus not initiated sexual activity during the study period) will be summarized as risk ratios or odds ratio with 95% confidence intervals for each study. Continuous outcomes such as number of sex partners will be summarized as

For peer review only

(unstandardized) mean differences and standard errors. In cases where sexual debut may be reported as a median age at sexual debut, published procedures for transforming median to mean and standard deviation to facilitate computation of relevant statistics will be used (59). If enough information is not provided to calculate an effect size, study authors will be contacted for clarification or to provide additional statistics. If the authors do not provide this information after one month, this study will be removed from the analysis but this effort will be reported.

If results of a repeated measure analysis are reported, authors will need to provide the correlation between pre-post measurements or provide enough information to calculate the correlation between measurements. If these statistics are not available, either in publication or after request, and the study was a controlled design, an effect size will be generated using post-intervention statistics provided groups are similar at baseline with respect to the outcome of interest and other relevant covariates.

Unit of analysis

Data will be extracted from each included study (unit of analysis) as follows. For dichotomous variables, number of participants in the intervention group and the number of participants in the standard/control group will be used. For continuous variables, the mean, standard deviation, and the number of participants in the intervention and control groups will be used. For studies with multiple intervention groups, each pair-wise comparison will be included separately. Moreover, for dichotomous outcomes, we will divide both the number of events and the total number of participants. For continuous outcomes, means and standard deviations will not be changed but will be divided by the total number of participants.

Assessment of heterogeneity

For peer review only - http://bmjopen.bmj.com/site/about/guidelines.xhtml

Heterogeneity among studies and between subgroups will be assessed using a Chi² test with a significance level at $p < 0.10$. The degree of heterogeneity among studies and between subgroups will be assessed using the I^2 statistic using the following guide: I^2 = 0% to 40% as heterogeneity that might not be important, I^2 = 30% to 60% as moderate heterogeneity, I^2 = 50% to 90% as substantial heterogeneity, and I^2 = 75% to 100% as considerable heterogeneity (60)

Assessment of reporting bias

Publication bias will be assessed if at least 10 studies are included in the review. A funnel plot will be used to assess the magnitude of reporting bias as per Cochrane guidelines.

Data synthesis

Data will be pooled using the random effects model according to Chapter 9 of the *Cochrane Handbook for Systematic Reviews of Interventions* (60). Dichotomous outcomes' data (e.g., number of participants who experience sexual debut) will be reported as risk ratios and continuous data (e.g., number of sexual partners) as mean differences. Depending on the different types of interventions identified, such as sexuality education, economic interventions or a combination of different approaches, we will conduct pooled effects for each type of interventions to examine which are more effective in delaying sexual debut. Additionally, and where possible, we will identify and consider conceptual groupings such RCTs and non-RCT studies while conducting the analysis. All analyses will be done at 95% confidence intervals. Data will be analysed using the statistical software Comprehensive Meta-Analysis(56) . In studies where the effects of clustering have not been taken into account, standard deviations will be adjusted for the design effect, using intra-class coefficients, if they are provided in the study reports, or alternatively using external estimates obtained from similar studies. Stratifications will be done by school level (primary or

For peer review only - http://bmjopen.bmj.com/site/about/guidelines.xhtml

secondary (or age, if applicable), grade level, instructor (e.g., teacher or peer), intervention type (e.g. behaviour change communication, study setting).

Other acceptable data synthesis methods such as those descried by McKenzie and Brennan (52) will be considered where meta-analysis is not possible. Such methods include summarizing effect estimates in case estimates of intervention effects are available but the variances of the effects are not reported or are not correct (52). Another method described by McKenzie and Brennan (52) is combining P values if no other information is available; differing statistical tests and outcomes across studies; or non-parametric test results reported. Lastly, vote counting based on the direction of the effect can be used when only the direction of effect is reported or there is no consistent effect measure or data reported across studies (52).

Findings will be organised in tables and charts, while presenting a description of the themes and concepts found in literature reflecting the review objectives. A summary narrative that synthesizes the information across tables and charts will be developed, critically highlighting the advances and gaps in research, with a focus to draw implications for future research.

Patient and public involvement

Patients and public will not be involved in the design and conduct of this review

Ethics and dissemination

Ethics approval is not required. The findings of this systematic review will be disseminated through peer-reviewed publications as well as in relevant stakeholders' fora. In case of any amendments to the protocol following its publication, the date of each amendment will be provided, change(s) described, and report the rationale for the change(s) in future publications arising from this protocol.

For peer review only - http://bmjopen.bmj.com/site/about/guidelines.xhtml

Author contribution: BWM, KJ, EKI and JO contributed to the conceptualization, and development of the protocol. BWM and KJ provided the initial draft of the protocol. HW and CWK provided a critical review of the protocol. All authors read and approved the final draft of the protocol.

Funding statement: Authors' time to develop this protocol was supported by the International Development Research Centre (Grant number 108676-002) and the Swedish International Development Cooperation Agency (Grant number 12103)

Competing interests: None declared

Patients consent for publication: Not required

For peer review only

References

1. Chandra-Mouli V, Camacho AV, Michaud PA. WHO guidelines on preventing early pregnancy and poor reproductive outcomes among adolescents in developing countries. *Journal of Adolescent Health*. 2013;52(5):517-22.
2. Nove A, Matthews Z, Neal S, Camacho AV. Maternal mortality in adolescents compared with women of other ages: Evidence from 144 countries. *The Lancet Global Health*. 2014;2(3):e155-64.
3. Santhya KG, Jejeebhoy SJ. Sexual and reproductive health and rights of adolescent girls: Evidence from low- and middle-income countries. *Global Public Health*. 2015;10(2):189-221.
4. Decker MR, Peitzmeier S, Olumide A, Acharya R, Ojengbede O, Covarrubias L, et al. Prevalence and health impact of intimate partner violence and non-partner sexual violence among female adolescents aged 15-19 years in vulnerable urban environments: A multi-country study. *Journal of Adolescent Health*. 2014;55(6 Suppl):S58-67.
5. Doyle AM, Mavedzenge SN, Plummer ML, Ross DA. The sexual behaviour of adolescents in sub-Saharan Africa: Patterns and trends from national surveys. *Tropical Medicine and International Health*. 2012;17:796-807.
6. Brahmbhatt H, Kågesten A, Emerson M, Decker MR, Olumide AO, Ojengbede O, et al. Prevalence and determinants of adolescent pregnancy in urban disadvantaged settings across five cities. *Journal of Adolescent Health*. 2014;55(6):S48-S57.
7. O'Donnell L, O'Donnell CR, Stueve A. Early sexual initiation and subsequent sex-related risks among urban minority youth: The reach for health study. *Family Planning Perspectives*. 2001:268-75.

For peer review only - http://bmjopen.bmj.com/site/about/guidelines.xhtml

8. Ahorlu CK, Pfeiffer C, Obrist B. Socio-cultural and economic factors influencing adolescents' resilience against the threat of teenage pregnancy: A cross-sectional survey in Accra, Ghana. *Reproductive Health*. 2015;12:117.

9. Ajayi AI, Okeke SR. Protective sexual behaviours among young adults in Nigeria: Influence of family support and living with both parents. *BMC Public Health*. 2019;19(1):983.

10. Bingenheimer JB, Asante E, Ahiadeke C. Peer influences on sexual activity among adolescents in Ghana. *Studies in Family Planning*. 2015;46(1):1-19.

11. Durowade KA, Babatunde OA, Omokanye LO, Elegbede OE, Ayodele LM, Adewoye KR, et al. Early sexual debut: prevalence and risk factors among secondary school students in Ido-ekiti, Ekiti state, South-West Nigeria. *African Health Sciences*. 2017;17(3):614-22.

12. Makenzius M, Larsson M. Early onset of sexual intercourse is an indicator for hazardous lifestyle and problematic life situation. *Scandinavian Journal of Caring Sciences*. 2013;27(1):20-6.

13. Peltzer K. Early sexual debut and associated factors among in-school adolescents in eight African countries. *Acta Paediatrica*. 2010;99(8):1242-7.

14. Louie KS, de Sanjose S, Diaz M, Castellsagué X, Herrero R, Meijer CJ, et al. Early age at first sexual intercourse and early pregnancy are risk factors for cervical cancer in developing countries. *British Journal of Cancer*. 2009;100(7):1191-7.

15. Plummer M, Peto J, Franceschi S. Time since first sexual intercourse and the risk of cervical cancer. *International Journal of Cancer*. 2012;130(11):2638-44.

16. Kirby D. The impact of schools and school programs upon adolescent sexual behavior. *Journal of Sex Research*. 2002;39(1):27-33.

For peer review only

For peer review only - http://bmjopen.bmj.com/site/about/guidelines.xhtml

389 17. Bay-Cheng LY. The trouble of teen sex: The construction of adolescent sexuality through school-based sexuality education. *Sex Education*. 2003;3(1):61-74.

391 18. Agardh A, Cantor-Graae E, Östergren P-O. Youth, sexual risk-taking behavior, and mental health: A study of university students in Uganda. *International Journal of Behavioral Medicine*. 2012;19(2):208-16.

394 19. Boislard M-AP, Dussault F, Brendgen M, Vitaro F. Internalizing and externalizing behaviors as predictors of sexual onset in early adolescence. *The Journal of Early Adolescence*. 2013.

397 20. Brown LK, Hadley W, Stewart A, Lescano C, Whiteley L, Donenberg G, et al. Psychiatric disorders and sexual risk among adolescents in mental health treatment. *Journal of Consulting and Clinical Psychology*. 2010;78(4):590-7.

400 21. Callahan MR, Tolman RM, Saunders DG. Adolescent dating violence victimization and psychological well-being. *Journal of Adolescent Research*. 2003;18(6):664-81.

402 22. Kabiru CW, Izugbara CO, Beguy D. The health and wellbeing of young people in sub-Saharan Africa: An under-researched area? *BMC International Health and Human Rights*. 2013;13(1):11.

405 23. Kleinert S, Horton R. Adolescent health and wellbeing: A key to a sustainable future. *The Lancet*. 2016;387(10036):2355-6.

407 24. Patton GC, Sawyer SM, Santelli JS, Ross DA, Afifi R, Allen NB, et al. Our future: A Lancet commission on adolescent health and wellbeing. *The Lancet*. 2016;387(10036):2423-78.

409 25. Rock A, Barrington C, Abdoulayi S, Tsoka M, Mvula P, Handa S. Social networks, social participation, and health among youth living in extreme poverty in rural Malawi. *Social Science & Medicine*. 2016;170:55-62.

For peer review only

For peer review only - http://bmjopen.bmj.com/site/about/guidelines.xhtml

26. Viner RM, Ozer EM, Denny S, Marmot M, Resnick M, Fatusi A, et al. Adolescence and the social determinants of health. *The Lancet*. 2012;379:1641-52.

27. Beguy D, Ndugwa R, Kabiru CW. Entry into motherhood among adolescent girls in two informal settlements in Nairobi, Kenya. *Journal of Biosocial Science*. 2013;45(6):721-42.

28. Gonçalves H, Machado EC, Soares ALG, Camargo-Figuera FA, Seering LM, Mesenburg MA, et al. Sexual initiation among adolescents (10 to 14 years old) and health behaviors. *Brazilian Journal of Epidemiology*. 2015;18:25-41.

29. Althabe F, Moore JL, Gibbons L, Berrueta M, Goudar SS, Chomba E, et al. Adverse maternal and perinatal outcomes in adolescent pregnancies: The Global Network's Maternal Newborn Health Registry study. *Reproductive Health*. 2015;12 Suppl 2(Suppl 2):S8.

30. Moraes AN, Likwa RN, Nzala SH. A retrospective analysis of adverse obstetric and perinatal outcomes in adolescent pregnancy: The case of Luapula Province, Zambia. *Maternal Health, Neonatology and Perinatology*. 2018;4(1):20.

31. Marvin-Dowle K, Kilner K, Burley VJ, Soltani H. Impact of adolescent age on maternal and neonatal outcomes in the Born in Bradford cohort. *BMJ Open*. 2018;8(3):e016258.

32. Güneş P. The role of maternal education in child health: Evidence from a compulsory schooling law. *Economics of Education Review*. 2015;47.

33. Mensch BS, Chuang EK, Melnikas AJ, Psaki SR. Evidence for causal links between education and maternal and child health: Systematic review. *Tropical Medicine and International Health*. 2019;24(5):504-22.

34. Prickett KC, Augustine JM. Maternal education and investments in children's health. *Journal of Marriage and Family*. 2016;78(1):7-25.

35. WHO. *Promoting health through schools. Global school health initiative*. WHO, 1996.

For peer review only

For peer review only - http://bmjopen.bmj.com/site/about/guidelines.xhtml

36. WHO. *Global Accelerated Action for the Health of Adolescents (AA-HA!): Guidance to support country implementation*. Geneva: World Health Organization; 2017.

37. Allensworth DD, Kolbe LJ. The Comprehensive School Health Program: Exploring an expanded concept. *Journal of School Health*. 1987;57(10):409-12.

38. Chirwa-Kambole E, Svanemyr J, Sandøy I, Hangoma P, Zulu JM. Acceptability of youth clubs focusing on comprehensive sexual and reproductive health education in rural Zambian schools: A case of Central Province. *BMC Health Services Research*. 2020;20(1):42.

39. Deschesnes M, Martin C, Hill AJ. Comprehensive approaches to school health promotion: How to achieve broader implementation? *Health Promotion International*. 2003;18(4):387-96.

40. Mason-Jones AJ, Sinclair D, Mathews C, Kagee A, Hillman A, Lombard C. School-based interventions for preventing HIV, sexually transmitted infections, and pregnancy in adolescents. *Cochrane Database of Systematic Reviews*. 2016(11).

41. Salam RA, Faqqah A, Sajjad N, Lassi ZS, Das JK, Kaufman M, et al. Improving adolescent sexual and reproductive health: A systematic review of potential interventions. *Journal of Adolescent Health*. 2016;59(4):S11-S28.

42. Denford S, Abraham C, Campbell R, Busse H. A comprehensive review of reviews of school-based interventions to improve sexual-health. *Health Psychology Review*. 2017;11(1):33-52.

43. Wang T, Lurie M, Govindasamy D, Mathews C. The effects of school-based condom availability programs (CAPs) on condom acquisition, use and sexual behavior: A systematic review. *AIDS and Behavior*. 2018;22(1):308-20.

For peer review only - http://bmjopen.bmj.com/site/about/guidelines.xhtml

44. Koo HP, Rose A, El-Khorazaty MN, Yao Q, Jenkins RR, Anderson KM, et al. Evaluation of a randomized intervention to delay sexual initiation among fifth-graders followed through the sixth grade. *Sex Education*. 2011;11(01):27-46.

45. Fonner VA, Armstrong KS, Kennedy CE, O'Reilly KR, Sweat MD. School based sex education and HIV prevention in low- and middle-income countries: A systematic review and meta-analysis. *PloS One*. 2014;9(3):e89692-e.

46. Mathews C, Aarø LE, Grimsrud A, Flisher AJ, Kaaya S, Onya H, et al. Effects of the SATZ teacher-led school HIV prevention programmes on adolescent sexual behaviour: Cluster randomised controlled trials in three sub-Saharan African sites. *International Health*. 2012;4(2):111-22.

47. Jemmott JB, Jemmott LS, O'Leary A, Ngwane Z, Icard LD, Bellamy SL, et al. School-based randomized controlled trial of an HIV/STD risk-reduction intervention for South African adolescents. *Archives of Pediatrics & Adolescent Medicine*. 2010;164(10):923-9.

48. Cowan FM, Pascoe SJ, Langhaug LF, Mavhu W, Chidiya S, Jaffar S, et al. The Regai Dzive Shiri Project: Results of a randomised trial of an HIV prevention intervention for Zimbabwean youth. *AIDS*. 2010;24(16):2541.

49. Maticka-Tyndale E, Wildish J, Gichuru M. Thirty-month quasi-experimental evaluation follow-up of a national primary school HIV intervention in Kenya. *Sex Education*. 2010;10(2):113-30.

50. Mmbaga EJ, Kajula L, Aarø LE, Kilonzo M, Wubs AG, Eggers SM, et al. Effect of the PREPARE intervention on sexual initiation and condom use among adolescents aged 12–14: A cluster randomised controlled trial in Dar es Salaam, Tanzania. *BMC Public Health*. 2017;17(1):322.

For peer review only - http://bmjopen.bmj.com/site/about/guidelines.xhtml

51. Moher D, Shamseer L, Clarke M, Ghersi D, Liberati A, Petticrew M, et al. Preferred reporting items for systematic review and meta-analysis protocols (PRISMA-P) 2015 statement. *Systematic reviews*. 2015;4(1):1.

52. McKenzie J, Brennan S. *Chapter 12: Synthesizing and presenting findings using other methods In: Higgins JPT, Thomas J, Chandler J, et al., eds Cochrane Handbook for systematic reviews of interventions*. 2019.

53. Declaration M, editor Preventing through education. First meeting of Ministers of Health and Education to Stop HIV and STIs in Latin America and the Caribbean (Mexico City: 1 August 2008); 2008.

54. Ministries of Education, Ministries of Health. *Ministerial commitment on comprehensive sexuality education and sexual and reproductive health services for adolescents and young people in Eastern and Southern Africa (ESA)*. Angola, Botswana, Burundi, Democratic Republic of Congo, Ethiopia, Kenya…; 2013.

55. Ouzzani M, Hammady H, Fedorowicz Z, Elmagarmid A. *Rayyan — a web and mobile app for systematic reviews*. Systematic Reviews; 2016.

56. Borenstein M, Rothstein H, Cohen J. Comprehensive meta-analysis: A computer program for research synthesis. *Englewood, NJ: Biostat*. 1999.

57. Higgins JP, Thomas J, Chandler J, Cumpston M, Li T, Page MJ, et al. *Cochrane handbook for systematic reviews of interventions*: John Wiley & Sons; 2019.

58. Viswanathan M, Berkman ND, Dryden DM, Hartling L. *Assessing risk of bias and confounding in observational studies of interventions or exposures: further development of the RTI item bank*. RTI International–University of North Carolina at Chapel Hill Evidence-

For peer review only

For peer review only - http://bmjopen.bmj.com/site/about/guidelines.xhtml

503 based Practice Center, Research Triangle Park, NC; The University of Alberta Evidence-
504 based Practice Center, Edmonton, Canada, 2013.
505 59. Hozo SP, Djulbegovic B, Hozo I. Estimating the mean and variance from the median, range,
506 and the size of a sample. *BMC Medical Research Methodology*. 2005;5(1):13.
507 60. Deeks JJ, Higgins JP, Altman DG, Group CSM. *Analysing data and undertaking*
508 *meta-analyses*: John Wiley & Sons; 2019. 241-84 p.

509

For peer review only

For peer review only - http://bmjopen.bmj.com/site/about/guidelines.xhtml

Word Count: 3,360 words (excludes the title page, abstract, and references

For peer review only

For peer review only - http://bmjopen.bmj.com/site/about/guidelines.xhtml

Search strategy

Pubmed

1. (intervention[Title/Abstract]) OR (program*[Title/Abstract])
2. (school[Title/Abstract]) OR (institution[Title/Abstract]) OR (academic[Title/Abstract]) OR (education[Title/Abstract])
3. (sexual debut[Title/Abstract]) OR (sexual initiation[Title/Abstract]) OR (sexual delay[Title/Abstract]) OR (sexual activity [Title/Abstract])
4. (adolesce*[Title/Abstract]) OR ("young people"[Title/Abstract]) OR (youth[Title/Abstract]) OR (teenage*[Title/Abstract]) OR (learner[Title/Abstract]) OR (children[Title/Abstract])
5. (Africa south of the Sahara [MeSH Terms]) OR (Africa [MeSH Terms])
6. ("2009/01/01"[Date - Publication]: "2020/12/31"[Date - Publication])
7. #1 AND #2 AND #3 AND #4 AND #5 AND #6

   ("intervention"[Title/Abstract] OR "program*"[Title/Abstract]) AND ("school"[Title/Abstract] OR "institution"[Title/Abstract] OR "academic"[Title/Abstract] OR "education"[Title/Abstract]) AND ("sexual debut"[Title/Abstract] OR "sexual initiation"[Title/Abstract] OR "sexual delay"[Title/Abstract] OR "sexual activity"[Title/Abstract]) AND ("adolesce*"[Title/Abstract] OR "young people"[Title/Abstract] OR "youth"[Title/Abstract] OR "teenage*"[Title/Abstract] OR "learner"[Title/Abstract] OR "children"[Title/Abstract]) AND ("africa south of the sahara"[MeSH Terms] OR "africa"[MeSH Terms]) AND 2009/01/01:2020/12/31[Date - Publication]

For peer review only - http://bmjopen.bmj.com/site/about/guidelines.xhtml

PRISMA-P 2015 Checklist

This checklist has been adapted for use with protocol submissions to *Systematic Reviews* from Table 3 in Moher D et al: Preferred reporting items for systematic review and meta-analysis protocols (PRISMA-P) 2015 statement. *Systematic Reviews* 2015 **4**:1

Section/topic	#	Checklist item	Information reported: Yes	Information reported: No	Line number(s)
**ADMINISTRATIVE INFORMATION**					
**Title**					
Identification	1a	Identify the report as a protocol of a systematic review	☒	☐	1 - 2
Update	1b	If the protocol is for an update of a previous systematic review, identify as such	☐	☒	Not applicable
**Registration**	2	If registered, provide the name of the registry (e.g., PROSPERO) and registration number in the Abstract	☐	☒	Not registered
**Authors**					
Contact	3a	Provide name, institutional affiliation, and e-mail address of all protocol authors; provide physical mailing address of corresponding author	☒	☐	6 - 15
Contributions	3b	Describe contributions of protocol authors and identify the guarantor of the review	☒	☐	447 - 450
**Amendments**	4	If the protocol represents an amendment of a previously completed or published protocol, identify as such and list changes; otherwise, state plan for documenting important protocol amendments	☒	☐	98 - 100
**Support**					
Sources	5a	Indicate sources of financial or other support for the review	☒	☐	451 - 452
Sponsor	5b	Provide name for the review funder and/or sponsor	☒	☐	451 - 452
Role of sponsor/funder	5c	Describe roles of funder(s), sponsor(s), and/or institution(s), if any, in developing the protocol	☒	☐	451 - 452
**INTRODUCTION**					
**Rationale**	6	Describe the rationale for the review in the context of what is already known	☒	☐	74 - 91
**Objectives**	7	Provide an explicit statement of the question(s) the review will address with reference to participants, interventions, comparators, and outcomes (PICO)	☒	☐	91 - 93

BioMed Central The Open Access Publisher

Section/topic	#	Checklist item	Information reported: Yes	Information reported: No	Line number(s)
**METHODS**					
**Eligibility criteria**	8	Specify the study characteristics (e.g., PICO, study design, setting, time frame) and report characteristics (e.g., years considered, language, publication status) to be used as criteria for eligibility for the review	☒	☐	95 - 134
**Information sources**	9	Describe all intended information sources (e.g., electronic databases, contact with study authors, trial registers, or other grey literature sources) with planned dates of coverage	☒	☐	135 - 151
**Search strategy**	10	Present draft of search strategy to be used for at least one electronic database, including planned limits, such that it could be repeated	☒	☐	152 - 161
STUDY RECORDS					
Data management	11a	Describe the mechanism(s) that will be used to manage records and data throughout the review	☒	☐	164 - 192
Selection process	11b	State the process that will be used for selecting studies (e.g., two independent reviewers) through each phase of the review (i.e., screening, eligibility, and inclusion in meta-analysis)	☒	☐	164 - 170
Data collection process	11c	Describe planned method of extracting data from reports (e.g., piloting forms, done independently, in duplicate), any processes for obtaining and confirming data from investigators	☒	☐	172 - 177
**Data items**	12	List and define all variables for which data will be sought (e.g., PICO items, funding sources), any pre-planned data assumptions and simplifications	☒	☐	177 - 192
**Outcomes and prioritization**	13	List and define all outcomes for which data will be sought, including prioritization of main and additional outcomes, with rationale	☒	☐	115 - 119
**Risk of bias in individual studies**	14	Describe anticipated methods for assessing risk of bias of individual studies, including whether this will be done at the outcome or study level, or both; state how this information will be used in data synthesis	☒	☐	193- 230
DATA					
**Synthesis**	15a	Describe criteria under which study data will be quantitatively synthesized	☒	☐	270 - 285
	15b	If data are appropriate for quantitative synthesis, describe planned summary measures, methods of handling data, and methods of combining data from studies, including any planned exploration of consistency (e.g., I^2, Kendall's tau)	☒	☐	235 - 265
	15c	Describe any proposed additional analyses (e.g., sensitivity or subgroup analyses, meta-regression)	☒	☐	275 – 277
	15d	If quantitative synthesis is not appropriate, describe the type of summary planned	☒	☐	282 - 285
**Meta-bias(es)**	16	Specify any planned assessment of meta-bias(es) (e.g., publication bias across studies, selective reporting within studies)	☒	☐	276 - 281
**Confidence in cumulative evidence**	17	Describe how the strength of the body of evidence will be assessed (e.g., GRADE)	☒	☐	231 - 234

BioMed Central
The Open Access Publisher

For peer review only

BioMed Central
The Open Access Publisher
